# Exploring Nurses’ Experiences of Autonomy in Intensive Care Units: A Qualitative Descriptive Study

**DOI:** 10.1155/jonm/1385907

**Published:** 2026-10-09

**Authors:** Amal S. Haji Assa, Sultan M. Alharthi, Fai H. Alqarni, Ahdab B. Mandili, Shahad A. Alnefaie, Suad A. Alharbi

**Affiliations:** ^1^ Clinical Nursing Practices Department Faculty of Nursing Umm Al-Qura University Makkah Saudi Arabia uqu.edu.sa; ^2^ Hematology Department King Abdullah Medical City Makkah Saudi Arabia kamc.med.sa; ^3^ Coronary Care Unit King Abdullah Medical City Makkah Saudi Arabia kamc.med.sa; ^4^ Cardiology Ward King Abdullah Medical City Makkah Saudi Arabia kamc.med.sa; ^5^ Emergency Department Al-Noor Specialist Hospital Makkah Saudi Arabia nsh.med.sa

**Keywords:** advanced nursing, autonomy, descriptive, experience, ICU, independence, nursing, policy, qualitative, scope of practice

## Abstract

**Background:**

Nursing autonomy is essential in intensive care units (ICUs). However, there is limited understanding of how nurses perceive autonomy within their practice.

**Aim:**

To explore nurses’ perception and experience of autonomy in ICUs.

**Design:**

A qualitative descriptive study.

**Methods:**

The study was conducted with 14 ICU nurses through interviews from May to August 2025. Nurses’ characteristics were described, and interviews were transcribed verbatim and analyzed using a primarily inductive, semantic codebook thematic analysis informed by Braun and Clarke’s six‐phase framework.

**Results:**

Seven main themes and 25 subthemes emerged from the interviews, revealing personal and organizational factors that influence nursing autonomy in ICUs. The themes were as follows: nursing autonomy’s definition and perception, clinical practices, facilitators, barriers, perceived influence on patient care, perceived influence on nurse personal and professional development, and future advancements.

**Conclusion:**

The findings provide insight into ICU nurses’ experiences and perceptions of autonomy. Nurses reported that autonomy was influenced by clinical competence, organizational support, and clear guidelines. Nurses perceived that independent nursing care and timely actions undertaken under approved protocols could support patient care, professional confidence, and job satisfaction. Limited decision‐making authority, inadequate staffing, and unclear institutional support were reported as barriers. Nursing autonomy could strengthen nurses’ confidence and job satisfaction, which nurses perceived as contributing to better patient care.

**Implications for Nursing Management:**

The findings can guide nurse managers in implementing structured competency‐based education, reinforcing clearly defined scopes of practice that are consistent with institutional policies, and adopting supportive leadership practices to promote nursing autonomy. Hospital administrators and national policymakers can further use the findings to advance nursing practice through developing organizational and regulatory frameworks required to authorize and safely support expanded autonomous practice.

## 1. Introduction

Professional autonomy is a cornerstone of professional practice, shaping a healthy work environment [1]. The complexity of the intensive care unit (ICU) environment and the critical condition of patients mandate that nurses practice autonomy [2]. Nursing autonomy refers to the ability to act based on clinical judgment, knowledge, ethical principles, and the full scope of practice [3]. Autonomy is influenced by personal skills, organizational policies, and interdisciplinary collaboration [4].

Autonomy can be applied in various nursing practices such as preventing patient falls, preventing skin injuries, performing emergency defibrillation, and managing sedation and intubation [5–8]. ICU nurses with greater levels of autonomy exhibit lower levels of job stress, depression, burnout, and intention to leave their jobs. Autonomy impacts nurses’ satisfaction and confidence, which contribute positively to creating a safe environment that optimizes patient outcomes in ICUs [3, 7, 9, 10].

Autonomy is limited by moral distress, blame culture, organizational management style, a lack of professional support, and absence of appropriate education [4, 11]. The lack of understanding of the concept of autonomy and the absence of protocols negatively impact nurses’ perception of their scope of practice [8, 11].

Existing evidence has consistently identified the factors shaping nursing autonomy, including education, clinical experience, managerial support, organizational policy, staffing, interdisciplinary hierarchy, and scope of practice restrictions. In Saudi critical care settings, autonomy has been examined primarily through quantitative assessment of its level and associated factors [5], while recent Saudi qualitative research has examined how organizational structures, protocols, and interprofessional hierarchies shape nurses’ decision‐making authority within interprofessional teams [12]. Less attention has been directed toward how bedside ICU nurses distinguish between independent nursing judgment, actions undertaken within approved protocols or standing orders, and practices that remain outside their formally authorized scope or institutional privileges.

This gap is particularly significant in Saudi tertiary ICUs, where protocol‐driven practice requires rapid clinical action while scope of practice regulation is being actively restructured under Vision 2030 [13]. This study therefore explores how ICU nurses experience and navigate the boundaries between independent nursing judgment, protocol‐authorized practice, and formally authorized scope of practice.

## 2. Methods

### 2.1. Aim

There are limited studies on how nurses in ICUs perceive the concept of autonomy within their practice. Therefore, we aim to explore how nurses in ICUs of three hospitals perceive and experience autonomy in their practice.

### 2.2. Design

A qualitative descriptive design was employed to provide a comprehensive description of nurses’ perception and experience of nursing autonomy in ICUs. This study aimed to obtain a rich understanding of participants’ experiences without seeking the essence of the phenomenon. The study adhered to the Standards for Reporting Qualitative Research (SRQR) to ensure methodological rigor, transparency, and comprehensiveness in reporting qualitative findings [14].

### 2.3. Study Setting and Sampling

The study was conducted across three tertiary hospitals in Makkah Region, Saudi Arabia. Hospitals 1 and 2 comprised one general ICU caring for medical‐surgical cases, while Hospital 3 comprised two ICUs, caring for medical‐surgical, cardiac, and trauma cases. Purposive sampling based on selection criteria was applied to select and recruit nurses working in adult ICUs. The selection criteria ensured that all participants had experience with the phenomenon [15]. Inclusion criteria were registered nurses (RNs) with over 6 months of independent ICU practice following completion of the institutional orientation and preceptorship program. Nurses working in non‐ICU settings, with less than 6 months of independent ICU practice, and who declined to participate were excluded. Nineteen eligible nurses were invited to participate. Fourteen nurses agreed to participate, yielding a response rate of 73.7%, while five declined, mainly because of time constraints.

### 2.4. Data Collection

Semistructured interviews were conducted between May and August 2025 by two researchers (Amal S. Haji Assa) and (Sultan M. Alharthi) who have backgrounds in critical care nursing and research. Researcher (Sultan M. Alharthi) organized visits to the settings to explain the study information. Participants were invited to attend virtual interviews, all conducted in English via the Webex platform, and they voluntarily participated. The interview duration ranged from 30 to 45 min. An interview protocol was followed to ensure consistency and rigor throughout the data collection process. Field notes of participant tone and contextual observations of the participants were recorded by the interviewer during each interview. All interviews were audio‐recorded using Webex. Four researchers shared responsibility for transcribing the interviews verbatim. Each transcript was independently cross‐checked against the corresponding audio recording by another researcher to ensure accuracy.

Informed consent was obtained, confirming participants’ agreement to participate and to be audio‐recorded, after providing descriptions of the study purpose and procedures. Sociodemographic data were collected, including age, gender, marital status, level of education, years of experience in hospitals and in the ICU, and job position. The interview questions were constructed as open‐ended questions to explore ICU nurses’ experiences and perceptions of autonomy, procedures performed autonomously, barriers and facilitators of autonomy, nurses’ perceptions of how autonomy influenced patient care and nurses’ professional experiences, and perspectives on the future development of autonomy. Participants were asked to clarify answers if needed and reflect on their statements to encourage conversation.

### 2.5. Data Analysis

Thematic analysis was conducted manually using Microsoft Word and Excel. Color coding was used to highlight potential codes and facilitate the development of themes. Sociodemographic data were analyzed using descriptive statistics, including frequencies and percentages. Qualitative data were analyzed using a primarily inductive, semantic codebook thematic analysis informed by Braun and Clarke’s six‐phase framework. An inductive semantic approach was used, as codes were developed reflecting participants’ experiences and perceptions of autonomy rather than from a predetermined coding framework, while allowing interpretation during the development of broader categories and themes. Preliminary analysis occurred concurrently with data collection. Two researchers independently familiarized themselves with the data by reading the transcripts line by line to gain an overall understanding and generated initial codes from significant statements. The researchers compared emerging codes from prior participants after each interview. Similar codes were grouped into broader categories to develop preliminary themes and subthemes. Two additional researchers independently reviewed the coding decisions and preliminary thematic structure against the original transcripts. Differences in interpretation were discussed and resolved through re‐examination of the transcripts. The themes and subthemes were reviewed by the principal investigator (PI) to ensure accuracy and coherence and were subsequently refined, defined, and organized into a thematic matrix. Finally, a composite description of the findings was developed, incorporating the final themes and subthemes. Following the 12th interview, no new codes or substantive themes emerged compared to the previous 11 interviews, indicating data saturation. Two additional interviews were conducted and analyzed to confirm saturation, matching the existing themes.

### 2.6. Ethical Considerations

The institutional review board (IRB) approval and institutional permissions were obtained, covering all three participating hospitals to ensure compliance with ethical principles throughout the research process. Participants were asked to voluntarily participate and to consent to their participation in the study and publication of the shared anonymized quotations. Following each Webex interview, the interview recording was assigned a numerical code, securely downloaded to a password‐protected computer accessible only to the research team, and deleted immediately from Webex. All recordings and transcripts were de‐identified and will be retained for 5 years after the study’s completion, and then will be permanently deleted, to maintain participants’ confidentiality and privacy.

### 2.7. Rigor and Reflexivity

To ensure credibility, multiple researchers reviewed the literature and developed the interview questions. Semistructured, in‐depth interviews were conducted to enable participants to describe their experiences in detail, while follow‐up questions were used to clarify meanings and obtain rich descriptions. Member checking was applied during the interview through real‐time clarification or to confirm the participants’ responses when ambiguity arose. The two primary interviewers had no authority or employment relationship with the participants as they were not employed at the participating hospitals, reducing the potential for social desirability bias. Before and after each interview, both interviewers, who had backgrounds in critical care nursing, documented their preexisting beliefs, clinical experiences, and assumptions related to nursing autonomy in ICUs. These reflexive notes were used to acknowledge how professional experiences could influence the data collection and interpretation. During the interviews, participants were encouraged to describe their experiences in their own words through open‐ended questions, while leading questions were avoided. Participants received the study information, purpose, confidentiality assurance, and were reassured that there were no right or wrong answers.

To enhance the analysis credibility and dependability, we applied several strategies to ensure a systematic and transparent analytical process. Before analysis, researchers checked the accuracy of the recorded interviews and verbatim transcriptions. Two researchers independently developed the initial codes, categories, and preliminary themes. Coding decisions and theme development processes were documented to ensure consistency, transparency, and dependability.

Emerging codes and themes were compared to the main transcripts regularly to support confirmability and to make sure that interpretations were based on the participants’ experiences, not on researchers’ assumptions. Differences in coding and interpretation were addressed through discussion and review of the original transcripts, followed by refinement of the codebook and thematic structure. Finally, the PI reviewed the final themes and subthemes for coherence with the dataset. To ensure transferability, we provided a thick description of the study’s methodology, participant characteristics, results, and discussion. Participants included nurses with varying levels of clinical experience and backgrounds. Detailed descriptions of the study enable readers to determine the applicability of the results to other critical care settings with similar organizational and clinical characteristics. Interview recordings, the analytic process, theme development notes, and documentation of methodological decisions were kept, enhancing the transparency and dependability of the research process.

## 3. Findings

### 3.1. Characteristics of Participants

Fourteen nurses from three hospitals participated. Data saturation was achieved by the 12th interview and confirmed by two additional interviews. Table 1 shows the participants’ characteristics. Participants’ ages varied from 24 to 44 years, with the majority being female (85.71%, *n* = 12). Nurses’ marital status was almost equally divided between single and married, while their educational level was similarly divided between bachelor’s and master’s/high diploma degrees. A total of 28.57% of nurses (*n* = 4) had 11–16 years of hospital experience, and 28.57% (*n* = 4) had 7–10 years of ICU experience. Nurses’ job positions were equally split between bedside nurses and charge nurses (42.86%, *n* = 6), while the remainder comprised one head nurse and one clinical instructor.

**TABLE 1:** Characteristics of participants (*n* = 14).

Characteristics	Categories	Frequency	Percentage (%)
Age	24–29	4	28.57
	30–34	3	21.43
	35–39	4	28.57
	40–44	3	21.43

Gender	Female	12	85.71
	Male	2	14.29

Marital status	Single	8	57.14
	Married	6	42.86

Level of education	Bachelor	8	57.14
	Master/high diploma	6	42.86

Total years of experience in hospitals	7 to 12 months	1	7.14
	1 to 3 years	2	14.29
	4 to 6 years	3	21.43
	7 to 10 years	1	7.14
	11 to 16 years	4	28.57
	17 to 20 years	3	21.43

Total years of experience in ICU	7 to 12 months	2	14.29
	1 to 3 years	1	7.14
	4 to 6 years	3	21.43
	7 to 10 years	4	28.57
	11 to 16 years	3	21.43
	17 to 20 years	1	7.14

Job position	Staff nurse	6	42.86
	Head nurse	1	7.14
	Charge nurse	6	42.86
	Clinical instructor	1	7.14

### 3.2. Thematic Analysis of Nursing Autonomy in ICUs

Seven themes emerged from the interviews, revealing personal and organizational factors that influence nursing autonomy in ICUs. Figure 1 summarizes the seven themes and corresponding subthemes of nursing autonomy in ICUs. The themes were as follows: definition and perception of nursing autonomy in ICUs, common clinical nursing practices of autonomy in ICUs, facilitators of nursing autonomy in ICUs, barriers to nursing autonomy in ICUs, perceived influence of nursing autonomy in ICUs on patient care, perceived influence of nursing autonomy in ICUs on nurses’ personal and professional development, and future advancements of nursing autonomy in ICUs. Table 2 illustrates the thematic analysis process. Participants were assigned anonymized codes using the format P (participant number) (site number), where “P” refers to the participant. Site 1 included four participants (P1.1, P2.1, P3.1, and P4.1), Site 2 included five participants (P1.2, P2.2, P3.2, P4.2, and P5.2), and Site 3 included five participants (P1.3, P2.3, P3.3, P4.3, and P5.3), for a total of 14 participants.

**FIGURE 1:**
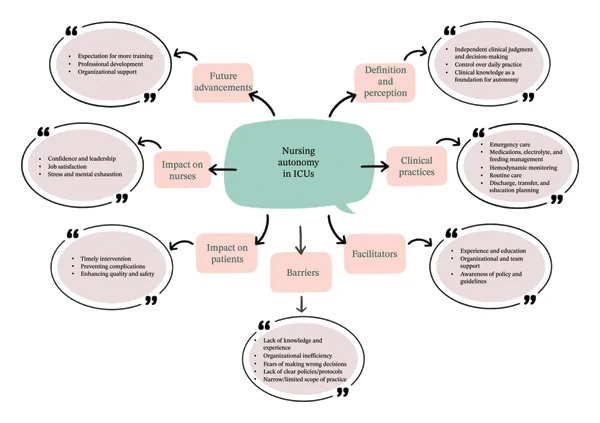
Thematic map illustrating the seven themes and their corresponding subthemes related to nursing autonomy in intensive care units, developed using Braun and Clarke’s six‐phase thematic analysis [18].

**TABLE 2:** Thematic analysis matrix of nursing autonomy in intensive care units (*n* = 14).

Theme	Description	Subtheme	Illustrative quotation example	Participants
1. Definition and perception of nursing autonomy in ICUs	The ability to make clinical decisions with minimal or no supervision from physicians or other healthcare professionals. It involves having the authority, confidence, and competence to carry out nursing interventions and make informed judgments. This level of independence requires comprehensive knowledge of the patient’s condition, treatment plan, and potential outcomes to ensure safe and effective care	Independent clinical judgment and decision‐making	“Ability of the nurse … hmmm … to make a decision alone without giving her advice …, yeah, without doctor orders.” P1.2 “The nurse’s ability in decision‐making by herself, depending on the evidence‐based … without referring to the supervisor or the senior staff.” P1.1 “The nurse’s ability to make independent decisions in practical procedures or in the ICU environment.” P2.3	P1.2, P2.2, P3.2, P4.2 P1.1, P2.1, P3.1, P4.1 P2.3, P4.3, P5.3
		Control over daily practice	“Having complete control and confidence to handle actions and decisions from the beginning of my shift until the end of my shift.” P3.2	P3.2
		Clinical knowledge as a foundation for autonomy	“To make a clinical decision in ICU, you should be able to know every single point about your patients, … decide the effect of the medication, the procedures, the prognosis of the patient’s disease … if you are able to know about that all, so … you can decide without refer to the supervisors or your seniors” P1.1 “Take clinical decision‐making or clinical decision based on her knowledge and experience, without going back to the specialist.” P3.1 “Nursing autonomy in ICU depends on your experience … is based on my knowledge and experience, and also the patient’s critical needs.” P3.3	P1.1, P3.1 P3.3

2. Common clinical nursing practices of autonomy in ICUs	Emergency and routine critical nursing interventions that are performed independently based on nurses’ clinical judgment, assessment, experience, and hospital protocols without requiring immediate orders from physicians, to respond to patient needs in real time to ensure safe and effective care	Emergency care: ‐ CPR and RRT ‐ Oxygen therapy ‐ Suctioning ‐ Mechanical ventilation ‐ CRRT	“When the patient is arrested … hmm. … immediately stop all the sedation and start CPR according to the ACLS protocol.” P3.2 “If the patient is arrested and the patient is on full code status, not on DNR status, immediately, I will start chest compression without informing the doctor, and another staff member will activate the code till the doctor comes…” P1.3 “I do these from the beginning of my shift, patients’ assessment, managing the patients, suctioning, titrating or decreasing oxygen.” P3.2 “For mechanical ventilator parameters, we can change some of the parameters … if my patient’s oxygen saturation is 100%, the patient is perfused well, and his capillary refill is very good, and less than 3 seconds, and he is on FiO2 100 percent. I will titrate gradually 90, 80, 70 to be accepted.” P1.3 “Maybe like a CRRT. As ICU nurses, it is fully dependent on us.” P4.2	P2.2, P3.2, P1.1, P2.1, P3.1 P1.3, P4.3, P5.3 P4.2
		Medications, electrolyte, and feeding management: ‐ Administration ‐ Termination ‐ Titration	“We can increase or decrease the Levophed and stop it without the doctor’s decision based on vital signs.” P2.2 “If the patient is on sedation and becoming agitated, I can titrate and increase the sedation.” P4.2 “…you can make a decision without referring to the supervisor. For example, if I want to start feeding, if I want to start to decrease a certain medication.” P1.1 “The patients may have an electrolyte imbalance. I can make the decision to treat the patient and correct the electrolytes.” P2.1 “When the patients have a high blood sugar level, according to the protocol in the hospital, we can start insulin infusion.” P3.1 “In a pulmonary edema patient, I already know the symptoms, so I have the right to take action to give Lasix according to the protocol …I will inform the doctor later.” P4.1 “I can titrate or increase the rate of inotropes based on MAP or blood pressure without confirming with the doctor.” P1.3	P1.2, P2.2, P3.2, P4.2, P5.2 P1.1, P2.1, P3.1, P4.1 P1.3, P2.3, P3.3, P5.3
		Hemodynamic Monitoring: ‐ Vital signs ‐ ECG ‐ Blood glucose	“If a patient has undergone a particular procedure, we will monitor BP and SpO2, to see whether they deteriorate.” P1.2 “When the patients have a high blood sugar level, according to the protocol or policies in the hospital, we can start insulin infusion.” P3.1	P1.2, P4.2 P1.1, P2.1, P3.1
		Routine care: ‐ Patient repositioning ‐ Restrain application ‐ Wound and pressure ulcer care	“And if the patient is stable enough to be repositioned, we will do that.” P1.2 “If I have an agitated patient, he will harm himself. We can restrain him first, then we will inform. Otherwise, he will remove the tube.” P5.2 “During bed making or patient bathing, the nurse assesses the patient’s skin. If there is a skin injury or a skin breakdown, meaning a bedsore, the nurse will assess the degree and the site of the bedsore and will apply a suitable dressing for it…” P1.3	P1.2 P1.1 P5.2 P3.1 P1.3
		Discharge, transfer, and education planning	“I had a patient ready for discharge. I checked the patient data, including cultures, and I found the patient was positive for MRSA. So, I stopped the transfer process, and the patient’s room was changed to a contact isolation room.” P2.3 “Educating the family and patients regarding the disease, care, medications, situation, and place is typically a nursing responsibility.” P3.1	P2.3 P3.1

3. Facilitators of nursing autonomy in ICUs	The conditions, resources, and supports that empower nurses to make independent clinical decisions and perform interventions confidently and competently, while ensuring safe and effective patient care	Experience and education	“I’m able to do a certain procedure or things autonomously, because I’m experienced enough to do those things.” P1.2 “Doing group discussion on inotropes, infection control, ICU bundles such as VAE, Sepsis, and Early mobilization bundles.” P5.2 “You should have advanced training, assessments, and practice all the critical issues in a simulation‐based learning environment.” P1.1 “For the new employees, we must have a good orientation program … staying at least 3 months working with preceptors … So, educational activities refresh all of our knowledge. Experience is not enough without knowledge. Knowledge is not enough without experience.” P1.3 “Having the knowledge, skills, and the attitude. You should have a strong understanding of pathophysiology, pharmacology, and critical care principles, which are essential for us as nurses.” P4.3	P1.2, P2.2, P4.2, P5.2, P1.1, P2.1, P3.1, P4.1 P1.3, P2.3, P3.3, P4.3, P5.3
		Organizational and team support	“Having clinical instructors is important to provide staff with updated evidence‐based practice protocols.” P4.2 “Having support from other healthcare professionals gives us confidence to intervene quickly when needed.” P4.2 “There should be real leadership and trust between the leaders and staff, and also for safety culture or safe environment practice.” P2.3 “Having enough staff.” P3.2 “You will be exhausted when you make a decision under pressure without enough support. If you don’t have good support to give you this opportunity or chance to make your own decision, of course, you will never be confident.” P1.1	P2.2, P3.2, P4.2 P2.1, P3.1 P1.3, P2.3, P3.3 P3.2, P1.1
		Awareness of policy and guidelines	“We start CPR according to the ACLS protocol.” P3.2 “Knowing the policies helps in my confidence to make clinical decisions.” P4.2 “We need clear protocols to empower the nurses to be decision makers.” P2.1 “We’ll answer with only one word, Policy!” P1.3	P2.2, P3.2, P4.2 P1.1, P2.1, P3.1 P1.3, P2.3

4. Barriers to nursing autonomy in ICUs	The internal and external factors that limit or restrict nurses’ ability to make independent clinical decisions and act autonomously in patient care. These barriers can affect confidence, quality of care, and early interventions in ICUs.	Lack of knowledge and experience	“If you are a new staff member in ICU, yes, we will find a lot of obstruction or a lot of barriers to learn … to make a critical decision.” P1.1 “Knowledge can also be a barrier. When I don’t have enough knowledge or am not knowledgeable enough to make this procedure or interventions, it can lead to negative patient outcomes.” P4.3	P1.2, P2.2, P4.2 P1.1, P2.1 P3.3, P4.3
		Organizational Inefficiency: ‐ Stress ‐ Workload ‐ Poor Communication ‐ Poor Teamwork ‐ Lack of Time Management	“Poor team dynamics, stress, and long‐term work will create pressure and could be a barrier to making a quick decision.” P2.2 “The culture of the doctors is that they cannot trust the nurse.” P2.1 “In the round, the nurses do not tell anything when presenting the case. She just keeps silent.” P2.1 “Barriers as the lack of doctors’ support, and the inadequate staff or work overload.” P5.3	P2.1, P3.1, P1.1, P2.1, P4.1 P3.3, P5.3
		Fear of making wrong decisions	“Sometimes we misuse autonomy and make wrong decisions in doing interventions or giving medications.” P5.2 “We fear making any decision.” P3.1	P5.2 P3.1
		Lack of clear policies and protocols	“We don’t have clear policies.” P4.2 “Lack of resources and … rigid policies prevent you from doing or you cannot do it.” P3.1 “If I have access to interfere with evidence‐based guidelines … it will help me in autonomy” P2.3	P2.2, P4.2, P5.2 P3.1 P2.3
		Narrow or limited scope of practice	“I have no privilege to have autonomy to do a central line, even if I’m qualified to do it. Not in my job description.” P5.2 “I am a nurse, I can do most procedures, but I don’t have the legal privilege to do these procedures, like catheter insertion, nasogastric tube insertion, CV line insertion, endotracheal tube insertion, but I don’t have the privilege.” P1.3 “In my nursing college, I trained to put in an NGT, but here I cannot.” P2.3 “The practice here is still narrow.” P3.3	P5.2 P1.3, P2.3, P3.3

5. Impact of nursing autonomy in ICUs on patient outcomes	The effects of the ability of ICU nurses to act independently based on assessments, clinical judgment, and real‐time interventions on patient recovery, safety, and care quality	Timely intervention	“Nurses can see the small changes that have happened to the patient so we can act immediately without waiting.” P3.2 “You will have a better outcome instead of waiting for a doctor to make a decision, especially in an emergency situation during the CPR.” P1.1 “The patients in intensive care are at a critical time, so the faster the nurse arrives, the sooner we can save them. This impacts both the patient and the care they receive.” P3.3	P1.2, P3.2, P4.2 P1.1, P3.1 P3.3
		Preventing complications	“If the patient is agitated, he will jump out of the bed and will self‐extubate if we don’t intervene in time.” P4.2 “If a patient has low blood pressure, what should I do? … In this situation, I must act, as this can definitely decrease mortality and morbidity rates.” P3.3 “Rapid interventions prevent deterioration, reducing rates of organ failure or cardiac arrest.” P5.3	P3.2, P4.2 P3.3, P5.3
		Enhancing quality of care and safety	“If the nurse is aware of the patient’s conditions and needs, she can develop a discharge plan that assures the continuity of care after discharge.” P2.2 “If we don’t know how to deal with an agitated patient effectively and autonomously, we will risk patient safety.” P4.2 “…fast decision‐making and early interventions save lives and improve the quality of care. Also, they enhanced patient safety, increased patient satisfaction, and improved the overall outcome.” P1.1 “It is a key determinant for patient satisfaction.” P4.3	P2.2, P4.2, P1.1, P2.1, P3.1 P1.3, P4.3

6. Impact of nursing autonomy in ICUs on nurse personal and professional growth	The effects of practicing autonomously in empowering or overwhelming ICU nurses to develop their confidence, critical thinking, leadership skills, and overall job satisfaction	Confidence and leadership	“To realize that I have done something positive for my patients and my profession, as well as increase my confidence level and job satisfaction.” P1.2 “Autonomy in practice will promote nurses’ confidence, critical thinking, and leadership skills.” P4.2 “It will increase my self‐esteem. It will create leaders.” P2.1 “Autonomy fosters a sense of confidence and self‐reliance in me.” P4.3	P1.2, P2.2, P3.2, P4.2, P5.2 P1.1, P2.1, P3.1 P3.3, P4.3, P5.3
		Job satisfaction	“To realize that I have done something positive for my patients and my profession, as well as increase my confidence level, job satisfaction, and feelings with the rest of my colleagues or other health teams.” P1.2 “Nurses’ satisfaction comes from knowing that they did everything for patients, which motivates them to give even more.” P2.2 “I feel prouder and more satisfied because I know I’m making a real difference for my patients.” P3.2 “I feel relaxed in my work area through the autonomy, so I will have the ability to give good outcomes for my patient, so I will do my work out of love.” P2.3	P1.2, P2.2, P3.2 P3.1 P1.3, P2.3, P4.3, P5.3
		Stress and mental exhaustion	“Working in stressful area, of course you will be exhausted especially when you make a decision under the pressure without enough support … if you don’t have good supports that give you this uh opportunity or chance to take your own decision, of course uh you will never be confident …” P1.1 “Some people cannot lead, cannot make decisions. They will be stressed if something goes wrong.” P2.1 “Sometimes it’s stressful if you don’t have any support … the staff will fear the legal if the patient is deteriorating for any reason.” P3.1	P1.1, P2.1, P3.1

7. Future advancements of nursing autonomy in ICUs	The aspirations of ICU nurses for enhanced autonomy to advance nursing practice and profession depending on both individual development and system/organizational support to expand the scope of practice and empower nurse‐led decision‐making	Expectation for more training	“Nurses will be more involved in the patient’s care plan if she has high autonomy. Her knowledge and skills levels will increase, and she will intervene with logic (reasoning skills)” P4.2 “If I educate my nurses well and train them well, for a long time, their autonomy will improve so that they will act with an evidence‐based background.” P2.3 “I believe nursing autonomy will expand with more authority to make a clinical decision, and nurses will be more integrated into multi‐disciplinary decision‐making.” P1.1 “The simulation training will help you and give you knowledge and skills to improve.” P3.1	P1.2, P2.2, P4.2 P1.3, P2.3 P1.1, P3.1, P4.1
		Professional development	“Adopting high and efficient technology such as artificial intelligence will facilitate autonomous decision‐making.” P2.2 “It will change the competencies. The competencies are sometimes limited, I think most of the ICU staff are more competent in some procedures. They do not have the privilege to do it.” P1.3 “It will increase the use of advanced practice nurses … nurses, practitioners, and clinical nurse specialists will increasingly be integrated into ICU teams… More ICUs will allow this role to diagnose, prescribe, and manage care independently.” P1.3 “The practice … is still narrow, and the policy and job description are general.” P3.3 “I can see nurses providing comprehensive primary and specialty care, including diagnosing, treating, and managing illness because. Nursing is evolving into an advanced profession.” P4.3	P2.2, P4.2 P1.3, P3.3, P4.3 P3.1, P4.1
		Organizational support	“Improvement in nursing autonomy only occurs with organizational support.” P3.2 “Also, if I give a clear scope of practice, like giving them a clear policy.” P2.3 “If I get support from my nurse manager to empower me, my autonomy will increase after 5 years.” P2.3 “The decision‐making process can be improved during the round. Instead of the nurse only taking orders from the doctor, she can have a role in the decision‐making process and have an effective role in this team.” P3.3	P3.2 P2.3, P3.3, P5.3 P2.1

*Note:* Participant codes are presented as “P” referring to participant (participant number) (site number). Site 1: P1.1, P2.1, P3.1, and P4.1 (*n* = 4); Site 2: P1.2, P2.2, P3.2, P4.2, and P5.2 (*n* = 5); Site 3: P1.3, P2.3, P3.3, P4.3, and P5.3 (*n* = 5).

#### 3.2.1. Theme 1: Definition and Perception of Nursing Autonomy in ICUs

Nurses shared varied perceptions of autonomy, which were categorized into three subthemes: independent clinical judgment and decision‐making toward patients’ care, control over daily practice, and clinical knowledge as a foundation for autonomy.

Independent clinical judgment and decision‐making: Nurses described autonomy as the ability to make clinical decisions and intervene immediately without direct supervision from other healthcare professionals. Independent nursing judgment was distinguished from actions requiring approved protocols or standing orders.

*“The nurse’s ability in decision-making by herself, depending on the evidence-based … but should be taken by herself without referring to the supervisor or the senior staff.”* P1.1

Autonomy was supported by the application of evidence‐based assessment and clinical judgment skills. Hence, autonomy is a skill that can be acquired through competence in practice and professional accountability.

Control over daily practice: Nursing autonomy can be shown as having control over nurses’ daily practice, reflecting their ability to assess, plan, prioritize, and manage tasks independently.

*“Having complete control and confidence to handle actions and decision from beginning of my shift until the end of my shift.”* P3.2

Having control over daily nursing care empowered nurses to take full responsibility for assessment, planning, prioritization, implementation, and evaluation, within their authorized scope, enhancing their confidence to carry out interventions independently. Interdependent treatment decisions remained subject to applicable orders and policies.

Clinical knowledge as a foundation for autonomy: Nurses emphasized that the level of independence requires comprehensive knowledge of the patient’s condition, treatment plan, and potential outcomes.

*“To make a clinical decision in ICU, you should be able to know all and every single point about your patients, … decide the effect of the medication, the procedures, the prognosis of the patient’s disease … if you are able to know about that all, so … you can decide without refer to the supervisor or your seniors.”* P1.1

The combination of knowledge and clinical judgment enables nurses to assess and intervene immediately to prevent complications, ensure safe care, and provide effective care.

#### 3.2.2. Theme 2: Common Clinical Nursing Practices of Autonomy in ICUs

Nurses described common clinical procedures that illustrate how they practice autonomy in ICUs. Clinical practices were categorized into five subthemes: emergency care, medication/electrolytes/feeding management, hemodynamic monitoring, routine care, and discharge/transfer/education planning. Although some of these procedures were performed independently, others were carried out following approved protocols or standing orders.

Emergency care: Nurses illustrated their ability to initiate cardiopulmonary resuscitation (CPR) and activate the Rapid Response Team as their essential roles guided by the Advanced Cardiac Life Support protocol, without seeking physicians’ orders.

*“When the patient’s arrested … hmm. … immediately stop all the sedation and start CPR according to the ACLS protocol.”* P3.2

Following evidence‐based protocols was crucial to patient survival, reflecting the importance of autonomous practice in emergencies. Table 2 illustrates other procedures that nurses reported performing in ICU settings based on their assigned responsibilities, validated competencies, and applicable institutional protocols or standing orders.

Medications, electrolytes, and feeding management: Nurses described administering, titrating, and discontinuing infusions and feeding based on their clinical assessment of the patient’s condition.

*“I can titrate or increase the rate of inotropes automatically based on MAP or blood pressure without confirming with the doctor.”* P1.3

As nurses described, titration of high‐alert medications based on patients’ status reflects autonomous clinical interventions through approved protocols and standing orders rather than independent prescribing. This practice requires advanced clinical competency, continuous education and training, and adherence to patient‐safety standards. Managing hemodynamically unstable patients in ICUs requires advanced clinical skills and the authority to make timely nursing decisions within the nurse’s authorized scope of practice.

Hemodynamic monitoring: Vital signs, cardiac, and blood sugar monitoring indicate the stability, improvement, or deterioration of the patient’s status. Hourly monitoring and assessment of patients in ICUs inform the nurse’s decision‐making and actions.

*“When the patients have a high blood sugar level, according to the protocol or policies in the hospital, we can start insulin infusion.”* P3.1

Continuous monitoring and assessment of patient status enabled nurses to apply clinical judgment and respond to changes in patients’ conditions. When treatment initiation was indicated, such as starting an insulin infusion, nurses acted under approved institutional protocols or policies rather than through independent prescribing authority.

Routine care: Nurses emphasized that their daily routine care is usually planned independently based on the patient’s needs. Procedures were performed following evidence‐based protocols to prevent complications.

*“During bed making, patient bathing, or patient care, the nurse assesses the patient’s skin. If there is a skin injury or a skin breakdown, meaning a bedsore, the nurse will assess the degree and the site of the bedsore and will apply a suitable dressing for it…”* P1.3

Applying independent nursing interventions and acting within approved evidence‐based protocols empowered nurses to practice clinical judgment to address patients’ actual problems or prevent potential complications.

Discharge, transfer, and education planning: Nurses mentioned their independent roles in planning patient discharge and transfer that included providing patient and family education.

*“I had a patient ready for discharge. I checked the patient data, including cultures, and I found the patient was positive for MRSA. So, I stopped the transfer process, and the patient’s room was changed to a contact isolation room.”* P2.3

Having the autonomy to intervene in critical situations was necessary to prevent serious complications and ensure patient safety.

#### 3.2.3. Theme 3: Facilitators of Nursing Autonomy in ICUs

Facilitators that support nurses’ autonomy in ICUs were categorized into three subthemes: experience/education, organizational and team support, and awareness of policy/guidelines. These subthemes emphasize the role of resources and support in promoting nurses’ autonomy.

Experience and education: Ongoing education through training, simulations, and workshops supports the development of knowledge. Experience and knowledge are interdependent and mutually reinforcing.

*“For the new employees, we must have a good orientation program … staying at least three months working with preceptors … So, educational activities refresh all of our knowledge. Experience is not enough without knowledge. Knowledge is not enough without experience.”* P1.3

Nurses highlighted the importance of comprehensive orientation and educational programs for newly recruited nurses to strengthen their scientific background and enhance nurses’ competence, which is a foundation for autonomous practice.

Organizational and team support: Nurses reported that trust from multidisciplinary and administrative teams builds their confidence and facilitates timely action, which is essential for maintaining a safe care environment.

*“There should be real leadership and trust between the leaders and staff, and also for safety culture or safe environment practice.”* P2.3

Close, trusting relationships with leaders and nurses could create a safe and supportive work environment, which can enhance nurses’ confidence in practicing autonomy in ICUs.

Awareness of policy and guidelines: Autonomy in nursing practice is facilitated by transparent policies and well‐established protocols.

*“Knowing the policies helps in my confidence to make clinical decisions.”* P4.2

Awareness of institutional policies can strengthen nurses’ confidence in making autonomous decisions.

#### 3.2.4. Theme 4: Barriers to Nursing Autonomy in ICUs

Barriers that limited nurses’ autonomy in ICUs were categorized into five subthemes: lack of knowledge or experience, organizational inefficiency, fears of making wrong decisions, lack of clear policies and protocols, and narrow or limited scope of practice. These barriers affect nursing decision‐making.

Lack of knowledge or experience: Nurses reported that while knowledge and experience facilitate clinical practice, they may also create obstacles for new nurses.

*“Knowledge can also be a barrier. When I don’t have enough knowledge or am not knowledgeable enough to make this procedure or interventions, it can lead to negative patient outcomes.”* P4.3

Insufficient knowledge of specific clinical interventions is a key barrier to autonomy, which can adversely affect patient outcomes.

Organizational inefficiency: Insufficient workplace environmental support, including ineffective teamwork and communication, can lead to stress, increased workload, and limited time, all of which adversely affect the quality and safety of patient care.

*“Poor team dynamics, stress, and long-term work will create pressure and could be a barrier to making a quick decision.”* P2.2

Long‐term work, stress, and poor team dynamics increase pressure on nurses, which can obstruct timely decision‐making.

Fears of making wrong decisions: Making decisions about procedures or interventions in patient’s care can raise fears or concern regarding their safety and validity.

*“Sometimes we misuse autonomy and make wrong decisions in doing interventions or giving medications.”* P5.2

Constant fear may increase the risk of decision‐making errors in patient care, which impacts the quality and safety of patient care, as well as the nurse’s confidence and autonomy in practice.

Lack of clear policies and protocols: A lack of resources, along with strict or unclear policies and protocols, can make nurses uncertain when making immediate decisions.

*“We don’t have clear policies.”* P4.2

Nurses indicated that ambiguity in policies and protocols negatively impacted their professional autonomy, as well as their independence and efficiency.

Narrow or limited scope of practice: The autonomy of nursing interventions depends on the defined scope of practice, although numerous interventions remain outside the job description or recognized scope of practice.

*“I am a nurse, I can do most procedures, but I don’t have the legal privilege to do these procedures, like catheter insertion, nasogastric tube insertion, CV line insertion, endotracheal tube insertion, but I don′t have the privilege.”* P1.3

Nurses perceived a mismatch between some skills they had acquired through education or training and the procedures they were institutionally or legally authorized to perform, limiting and restricting professional autonomy. Expanding the scope of practice is key to determining the degree of nurses’ professional autonomy.

#### 3.2.5. Theme 5: Perceived Influence of Nursing Autonomy in ICUs on Patient Care

Nurses discussed their perception of how autonomy influenced patient care. These perceived effects were categorized into three subthemes: timely intervention, preventing complications, and enhancing patients’ quality of care and safety.

Timely intervention: Nurses discussed how rapid and independent interventions can positively impact the patient’s journey during hospitalization.

*“The patients in intensive care are at a critical time, so the faster the nurse arrives, the sooner we can save them. This impacts both the patient and the care they receive.”* (P3.3)

Participants perceived that acting independently and promptly could support patient safety and quality of care, which they believed reflected the value of autonomous clinical judgment for patient outcomes.

Preventing complications: Nurses emphasized their daily routines and applied critical thinking to prevent adverse consequences or side effects in critically ill patients.

*“If a patient has low blood pressure, what should I do? … In this situation, I must act, as this can definitely decrease mortality and morbidity rates.”* (P3.3)

Participants believed that continuous assessment and timely intervention could help prevent or minimize complications and adverse outcomes while empowering nurses to practice independently within their authorized scope of practice.

Enhancing patients’ quality of care and safety: Nurses advocated that safety was a critical concern and the top priority for patients in ICUs.

*“…fast decision-making and early interventions save lives and improve the quality of care. Also, they enhanced patient safety, increased patient satisfaction, and improved the overall outcome.”* (P1.1)

Participants perceived that autonomous practice enabled them to exercise rapid clinical judgment and assume responsibility as patient advocates, which they perceived as supporting patient outcomes.

#### 3.2.6. Theme 6: Perceived Influence of Nursing Autonomy in ICUs on Nurse Personal and Professional Development

Nurses reported that practicing autonomy contributed to their personal and professional development, enhancing their confidence, critical thinking, leadership skills, and job satisfaction. These perceived impacts were categorized into three subthemes: confidence and leadership skills, job satisfaction, and stress and mental exhaustion.

Confidence and leadership skills: Nurses reported that the exercise of autonomy and the application of clinical judgment within ICUs significantly reinforced their professional competencies and elevated their self‐confidence.

*“Autonomy in practice will promote nurses’ confidence, critical thinking, and leadership skills.”* (P4.2)

Participants perceived autonomy as contributing to their competence, fostering their personal growth, and advancing their professional development. Practicing autonomy is important as it reinforces nurses’ confidence, strengthens their professional abilities, and cultivates leadership qualities.

Job satisfaction: Nurses discussed that autonomy plays a vital role in an empowering work environment, which contributes to higher levels of workplace satisfaction and a stronger sense of professional fulfillment.

*“To realize that I have done something positive for my patients and my profession, as well as increase my confidence level, job satisfaction, and feelings with the rest of my colleagues or other health teams.”* (P1.2)

Participants believed that their involvement in patient care and opportunities to practice autonomy could strengthen their self‐esteem, critical thinking skills, and their understanding of the patient’s condition, leading to higher levels of job satisfaction.

Stress and mental exhaustion: Autonomy was also associated with stress and mental exhaustion, highlighting the challenges and demands of independent decision‐making in high‐acuity clinical settings.

*“Working in stressful area, of course you will be exhausted especially when you make a decision under the pressure without enough support … if you don′t have good supports that give you this uh opportunity or chance to take your own decision, of course uh you will never be confident …”* (P1.1)

Making decisions under pressure in stressful environments can increase nurses’ exhaustion. Applying autonomy must be carefully supported to ensure effective practice and well‐being.

#### 3.2.7. Theme 7: Future Advancements of Nursing Autonomy in ICUs

Nurses highlighted their future aspirations to enhance autonomy in ICUs. Their expectations were categorized into three subthemes: expectation for more training, professional development, and organizational support. Future developments depend on individual growth and organizational support.

Expectation for more training: Nurses indicated that their future expectations include increased training opportunities to develop knowledge and skills.

*“If I educate my nurses well and train them well, for a long time, their autonomy will improve so that they will act with an evidence-based background.”* P2.3

Ongoing professional training over time strengthens nurses’ autonomy and supports practice grounded in evidence‐based practice.

Professional development: Professional development can be expanded through contributions from technology and artificial intelligence, alongside initiatives to enhance competencies and the scope of practice.

*“It will increase the use of advanced practice nurses … nurses, practitioners, and clinical nurse specialists will increasingly be integrated into ICU teams… More ICUs will allow this role to diagnose, prescribe, and manage care independently.”* P1.3

Nurses expressed aspirations for expanded roles that would enable appropriately qualified and credentialed nurses to apply advanced skills and contribute to diagnosis and independent management of care within an authorized advanced practice scope.

Organizational support: Healthcare institutions and administrations play an essential role in empowering and sustaining nursing independence.

*“If I get support from my nurse manager to empower me, my autonomy will increase after five years.”* (P2.3)

Healthcare organizations should provide strong managerial and organizational support to enhance nurses’ sense of autonomy and ensure a positive work environment.

## 4. Discussion

This study explored the experiences of nurses regarding their perception and practice of autonomy in ICUs. Nurses reported that autonomy in the ICU was affected by nurses’ personal skills, the culture of the organization, and support from the institution. Autonomy was perceived as the capacity to make independent decisions, combined with professional knowledge, confidence, and commitment to evidence‐based practice. This aligns with previous studies that emphasize autonomy as a foundation of professional nursing practice and a determinant of care quality [7, 11].

While several factors identified in this study are consistent with previous literature, our findings focus more on how bedside ICU nurses differentiated the limitations of autonomy in practice. Participants distinguished between independent nursing judgment, actions undertaken within approved protocols or standing orders, and practices for which they perceived clinical competence but lacked formal scope or institutional privileges. This distinction extends existing evidence by showing that ICU nursing autonomy is shaped not only by the degree of decision‐making independence but also by the source and formal boundaries of the authority under which nurses act.

Nurses described practicing autonomy during emergency situations and reported initiating life‐saving procedures such as CPR, adjusting ventilator settings, and titrating high‐alert medications according to institutional protocols and evidence‐based guidelines. However, these findings should be interpreted as participants’ reported experiences within their clinical settings rather than evidence of unrestricted independent practice. These findings were consistent with studies describing ICU nurses as first responders due to their continuous bedside presence, which may support timely intervention and help prevent complications [5, 6, 8].

Nurses also indicated that autonomy is often influenced by hospital policies and culture. Participants felt more confident when guided by clear, evidence‐based frameworks. This aligns with Hendriks et al. [16], who emphasized the importance of a supportive ICU environment that integrates protocols to empower nurses to make their own medical decisions. Conversely, unclear or overly restrictive policies led to hesitation and reduced independent decision‐making [11].

Nurses perceived clinical experience and education as important facilitators of autonomy. They described long experience, postgraduate education, and continuing professional development as factors that strengthened their knowledge, competence, and confidence in clinical decision‐making compared to newly graduated nurses. This finding aligns with a scoping review finding that nursing autonomy is built on clinical competence and decision‐making skills, evolving through clinical experience and continuing education [4]. Participants also emphasized the importance of simulation, continuing education, and hands‐on training in ensuring that ICU nurses remain updated, competent, confident, and able to make independent decisions.

Nurses reported that trust from managers and collaboration with a multidisciplinary team enhanced their ability to make independent decisions. Conversely, heavy workload, inadequate staffing, and poor communication increased stress and diminished confidence. Abbas and AL‐Fayyadh [9], Panunto and Guirardello [10], and Taleghani et al. [3] found similar results, illustrating that a supportive environment facilitates autonomy and decreases burnout. Leadership style and institutional culture play crucial roles in shaping how autonomy is perceived within ICUs.

Some nurses reported that autonomy was associated with stress and mental fatigue in their experiences. Participants reported the emotional burden of making decisions in high‐risk cases. This duality is consistent with previous studies, which found that autonomy can create psychological pressure in the absence of institutional support. Autonomy can simultaneously motivate and overwhelm nurses [3, 5, 9, 10, 17]. Therefore, autonomy should be supported by mentorship, clear protocols, and shared responsibility.

Another barrier was the limited scope of practice. Participants reported a gap between the skills learned in university and what they were allowed to perform in daily practice. Participants reported a perceived gap between procedures learned during their education or training and those they were legally and institutionally authorized and credentialed to perform in practice. Even nurses with advanced practice qualifications reported experiencing the same barriers as RNs because of restricted institutional policies. These findings are supported by studies that found restrictive organizational policies and limited decision‐making authority prevented nurses from applying their competence in patient care [3, 5, 9, 10, 17].

Finally, participants expressed strong expectations for advancing nursing autonomy through expanding the scope of practice and continuous education. Nurses anticipated future expanded roles similar to advanced practice nursing roles, including diagnosing, prescribing, and independent management of care. These goals emphasize empowering and advancing healthcare workers. Policy reforms, regulatory authorization, credentialing, competency‐based training, and supportive leadership are essential to transforming these aspirations into sustainable professional standards [13].

This study has several strengths. The qualitative descriptive methodology was appropriately selected, permitting an in‐depth exploration of nurses’ experiences of autonomy. The sample provided various descriptions of autonomy among ICU nurses with different levels of education, experience, and position across the three hospitals. Methodological transparency was implemented through detailed reporting of data collection and analysis processes. The study findings were contextually situated within three hospitals in one region governed by a single health administration. Hence, unique organizational and cultural factors in these hospitals may have influenced nurses’ perceptions and practices. Therefore, the transferability and contextual applicability of the study findings to other hospitals should be assessed by considering the similarities in organizational policies, nursing scopes of practice, and ICU environments.

## 5. Implications for Nursing Management

Based on nurses’ reported perceptions of how autonomy influences their practice and professional outcomes, several recommendations emerge from the findings for nurse managers, leaders, hospital administrators, and policymakers to strengthen and safeguard autonomous nursing practices in ICUs. Translating these findings into practice requires distinct actions from each healthcare decision‐maker. Each recommendation described as follows is grounded in the study themes.

Nurse managers, in collaboration with multidisciplinary clinical teams, can contribute to the development and implementation of standardized ICU protocols. Formal approval of protocols governing advanced clinical practices and authorization of expanded institutional privileges should occur through appropriate hospital credentialing and remain consistent with national regulations. This recommendation is supported by participants’ reflection that the clarity of institutional policies enhanced their confidence in autonomous practice (Themes 3 and 4). Also, participants emphasized knowledge and clinical judgment as the basis for autonomy, rather than years in practice (Theme 1). ICU leaders should support competency‐based evaluation and competency validation so that nurses can demonstrate readiness to perform responsibilities already authorized within their scope and institutional privileges.

Workload pressure, inadequate team dynamics, and long shift hours were identified as barriers to autonomy, contributing to hesitation and wrong decision‐making (Themes 4 and 6). Hospital administrators should align nurse‐to‐patient ratios in ICUs with national benchmarks, monitor workload, and address understaffing as a patient safety concern. In addition, participants described managerial trust as a direct facilitator of timely independent action (Themes 3 and 7). Nurse managers should support competent clinical decision‐making that relies on clinical judgment and remain available for consultation.

Participants linked structured orientation, preceptorship, and continuing education to autonomy (Themes 3 and 7). Hospitals should sustain formal preceptorship programs for ICU nurses, integrate simulation, and re‐evaluate competency periodically. Autonomy also should be integrated within interprofessional practice, and reported hesitation was found in contexts of weak nurse–physician communication (Themes 3 and 7). ICU leaders should promote nurses’ participation in multidisciplinary rounds, ensuring nurses discuss assessment findings and clinical recommendations rather than only reporting completed tasks. Structured communication tools such as SBAR should be embedded in handover and physician consultation. Nurse managers and administrators should foster psychologically safe environments where nurses can express uncertainty, discuss orders, and report errors without fear of blame, through debriefings after each critical incident (Themes 4 and 6). For policymakers, the findings support reviewing and expanding the scope of ICU nursing practice so that authorized practice aligns with nurses’ validated competencies (Themes 4 and 7). Such change requires action by national regulatory and professional associations rather than by institutional or managerial decision alone. These implications reflect nurses’ perceived experiences regarding autonomy practices in ICUs. Future research with objective outcome measures is needed to validate these perceptions.

## 6. Conclusion

This qualitative study provided a comprehensive description of ICU nurses’ perceptions and reported experiences of autonomy in their clinical practices. Although nurses reported providing critical care autonomously, unclear policy, fear of legal consequences, and a narrow scope of practice restricted their ability to work independently. Nurses believed that autonomy could not flourish without a trusting organization, effective communication, and clear organizational policies.

## Author Contributions

All listed authors meet the authorship criteria of the *Journal of Nursing Management*. Each author has made substantial contributions to the work. Amal S. Haji Assa and Sultan M. Alharthi: conceptualization, methodology, investigation, validation, writing–original draft, and reviewing and editing the manuscript. Fai H. Alqarni and Ahdab B. Mandili: conceptualization, methodology, investigation, formal analysis, validation, writing–original draft, and reviewing. Shahad A. Alnefaie and Suad A. Alharbi: conceptualization, methodology, investigation, formal analysis, validation, and reviewing.

## Funding

This research work was funded by Umm Al‐Qura University, Saudi Arabia, under grant number: 26UQU4330130GSSR02. The funding was awarded after completion of the study to support publication.

## Disclosure

All authors critically reviewed and approved the final version of the manuscript and agree to be accountable for the work.

## Ethics Statement

The institutional review board (IRB) approval and institutional permissions were obtained from the Ministry of Health (MOH) branch in Makkah Region, Saudi Arabia, covering all three participating hospitals (Approval No. H‐02‐K‐076‐0325‐1305).

## Conflicts of Interest

The authors declare no conflicts of interest.

## Data Availability

The datasets collected and analyzed in the study are available from the corresponding author upon request.
